# Association of Oxidative Stress Markers with Incident Hyperglycemia in Gestational Diabetes Mellitus in an Educational Intervention

**DOI:** 10.3390/nu17040680

**Published:** 2025-02-14

**Authors:** Mónica L. Ruiz-Martínez, Rita A. Gómez-Díaz, Adriana Leticia Valdez González, Selene Ángeles Mejía, Rafael Mondragón González, Margarita Díaz Flores, Ricardo César Saldaña Espinoza, Luz Angélica Ramírez-García, Mary Flor Díaz Velázquez, Niels H. Wacher

**Affiliations:** 1 Unidad de Investigación Médica en Epidemiología Clínica UMAE HE CMN SXXI, Instituto Mexicano del Seguro Social (IMSS), Av. Cuauhtemoc 330, Mexico City 06720, Mexico; ausie23@gmail.com (M.L.R.-M.); valdezlety@yahoo.com.mx (A.L.V.G.); selene1983bc@gmail.com (S.Á.M.); rafmg@hotmail.com (R.M.G.); ricesaes@gmail.com (R.C.S.E.);; 2 Servicio de Endocrinología, Unidad Médica de Alta Especialidad, Hospital de Gineco-Obstetricia No. 4 “Luis Castelazo Ayala”, Instituto Mexicano del Seguro Social (IMSS), Rio de la Magdalena 289, Mexico City 01090, Mexico; 3 Unidad Médica de Alta Especialidad, Hospital de Gineco-Obstetricia No. 3, Centro Médico Nacional “La Raza”, Dr. Víctor Manuel Espinosa de los Reyes Sánchez, Instituto Mexicano del Seguro Social (IMSS), Eje vial 1 Poniente, Mexico City 02990, Mexico

**Keywords:** oxidative stress markers, incident hyperglycemia, gestational diabetes mellitus

## Abstract

Background/Objective: This study is aimed to assess the link between oxidative stress markers and incident hyperglycemia in women with gestational diabetes mellitus (GDM) during an educational intervention. Methods: The study used a prospective cohort. Pregnant women with GDM who were 18–40 years old (*n* = 201) participated in an 18-month postpartum educational intervention emphasizing healthy practices (nutrition, breastfeeding, physical activity, and psychosocial support). GDM women were tested with an oral glucose tolerance test (OGTT) after the third month postpartum, and were classified as follows: (1) incident hyperglycemia (IHypergly) (*n* = 86) if they had a fasting plasma glucose (FPG) of 100–125 mg/dL, and impaired glucose tolerance of 2 h (140–199 mg/dL), or type 2 diabetes (T2D) with an FPG ≥ 126 or ≥200 mg/dL (2 h); and (2) without incident hyperglycemia (*n* = 115) if they had an FPG < 100 mg/dL and <140 mg/dL 2 h post-OGTT. Participants were evaluated at the end of pregnancy and post-intervention. Clinical, biochemical, anthropometric, dietary, and oxidative stress markers data (malondialdehyde, reduced glutathione, antioxidant capacity, carbonylated proteins, and adiponectin) were recorded. Multivariate logistic regression analysis identified an association between oxidative stress markers and incident hyperglycemia in women with GDM. Results: A total of 6% progressed to T2D, and 36.8% to prediabetes. At baseline, Ihypergly women exhibited elevated oxidative stress markers and adiponectin, and lower antioxidant capacity. Post-intervention, they showed higher antioxidant capacity, GSH, and adiponectin, and lower MDA. Basal malondialdehyde, pregestational BMI, HbA1c, and sugary food consumption positively correlated with Ihypergly. A high intake of antioxidants inversely correlated with incident hyperglycemia. Conclusions: Higher concentrations of plasma markers of oxidative stress are associated with postpartum incident hyperglycemia in women with gestational diabetes.

## 1. Background

Gestational diabetes mellitus (GDM) is defined as any degree of glucose intolerance that was first recognized during pregnancy. The severity of hyperglycemia is clinically important regarding both short- and long-term maternal and fetal risks [1]. According to the International Diabetes Federation, the prevalence of GDM is 14.2% worldwide, and 13.3% in Mexico [2].

Obesity is a major health problem in Mexico; it confers a great risk for developing type 2 diabetes (T2D). The National Survey of Health and Nutrition of 2022 reported a prevalence of 68% for overweight and obese individuals; 70.9% for abdominal adiposity, and 6.1% for T2D in women between 20 and 39 years of age [3,4].

During pregnancy, oxidative stress (a persistent imbalance between reactive oxygen species production and the activation of antioxidant systems) is enhanced, and it is implicated in embryo development, implantation, angiogenesis, placental development and function, fetal development, and labor. Nonetheless, in women with GDM, the hyperglycemic environment is associated with heightened levels of oxidative stress due to the overproduction of free radicals, placental oxidation reactions, and/or a defect in antioxidant defenses. Excessive oxidative stress can lead to massive cellular damage [5]. When oxidative stress increases, it promotes insulin secretion alterations, pancreatic ß-cell damage, and apoptosis [6]. Lipid peroxidation leads to the production of malondialdehyde (MDA), a stable, toxic, and reactive aldehyde. Proteins can be oxidized leading to an irreversible process (i.e., carbonylated proteins [5]).

Due to the necessity to overcome free radicals, organisms have developed a series of defense mechanisms [7]. Glutathione is present in high concentrations, so it is considered one of the most important cellular antioxidants. It exists in its reduced (GSH) or oxidized (GSSG) state [5].

It has been reported that women with GDM, in comparison to women without GDM, have increased maternal circulating levels of oxidative stress markers (MDA, and carbonylated proteins) and a lowered antioxidant defense (GSH) [8,9].

Inflammation and oxidative stress are involved in the development of GDM and are related since reactive oxygen species (ROS) can activate inflammatory cells and enhance the production of inflammatory mediators. Inflammation can lead to increased ROS release, causing a vicious cycle. Proinflammatory cytokines are up-regulated in women with GDM due to hyperglycemia and an increase in adiposity [10]. Adiponectin, an anti-inflammatory cytokine, could be considered as a potential marker for GDM since it takes part in energy homeostasis, has insulin-sensitizing properties, and has beneficial effects for oxidative stress [11]. Hypoadiponectinemia has been reported in women with GDM [12].

GDM poses a significant risk for developing T2D. In a systematic review and meta-analysis, the risk of developing T2D after GDM increased linearly with the duration of follow-up. The increments per year of follow-up were estimated at 9.6% [13]. Risk factors for the progression of T2D after a history of GDM are pre-pregnancy obesity, genetic predisposition to T2D, insulin treatment during pregnancy, postpartum weight gain, having an unfavorable diet, and performing low levels of physical activity [14].

In a systematic review (*n* = 4090) aimed to prevent T2D in people with impaired glucose tolerance or at high risk for T2D, the overall risk reduction of T2D was 0.53 (95% CI 0.41; 0.67) by having lifestyle interventions (weight reduction, increase physical activity, and eating a healthy diet with lower total fat and saturated fat intake, low consumption of sugar, and a high fiber intake) [15].

The early initiation of lifestyle interventions postpartum (dietary modification, weight loss, and increased physical activity) in women with a history of GDM has been effective in preventing T2D [16,17,18].

However, the role of oxidative stress in GDM, and its association with incident diabetes, remains unclear. This study aimed to assess the link between oxidative stress markers and incident hyperglycemia in women with GDM in an educational intervention.

## 2. Methods

A prospective cohort of pregnant women with GDM (*n* = 201) between 18 and 40 years of age was used. All women participated in an 18-month postpartum prevention program for T2D and obesity that emphasized healthy practices. It consisted of an educational intervention (nutritional education, physical activity, promoting breastfeeding, and offering psychosocial support). It included monthly postpartum and individualized visits of 1 h with a general practitioner and a nutritionist for the first 3 months and every 3 months after (visits 6, 9, 12, and 18). Videos and short messages were sent via mobile phone on a weekly basis with information promoting healthy lifestyle changes. The nutritional education included individualized meal plans promoting weight loss, a high-quality diet, and adequate portion sizes. The physical activity component consisted of structured and individualized exercises, gradually increasing in intensity over time.

All women were tested with an oral glucose tolerance test (OGTT) with 75 g glucose after the third month postpartum, and were classified as follows: (1) incident hyperglycemia (IHypergly) if they had an impaired fasting plasma glucose (FPG) of 100–125 mg/dL, an impaired glucose tolerance of 2 h (140–199 mg/dL), and T2D with an FPG ≥ 126 or ≥200 mg/dL (2 h); and (2) without incident hyperglycemia (wIHypergly) if they had an FPG < 100 mg/dL and <140 mg/dL at 2 h post-OGTT.

All participants were measured for glucose, triglycerides, LDL-cholesterol, insulin, and HbA1c with standard techniques and equipment, and clinical, anthropometric, diet, and lifestyle parameters. The International Physical Activity Questionnaire (IPAQ) was applied. It consists of recall, over the last seven days, of time dedicated to vigorous and moderate activity, and of walking and sitting [19]. For the purposes of the present study, physical activity was classified as inactive or active.

To determine the dietary parameters at baseline, a food frequency questionnaire (FFQ), standardized and validated for the Mexican population, was used. Quantification of nutrients was made with SNUT nutritional analysis software developed and validated by the National Institute of Public Health (INSP). The participants were asked about a list of 104 foods, products, and ingredients frequently consumed in Mexico in the last year, and they had to choose between ten options: (0) never, (1) less than once a month, (2) one to three times a month, (3) once a week, (4) two to four times a week, (5) five to six times a week, (6) once a day, (7) two to three times a day, (8) four to five times a day, and (9) six or more times a day. Foods, products, and ingredients with sugary content were placed into one group (fruit paste, orange juice, honey, marmalade, and flavored water), and their frequency intake was averaged, and then categorized into two groups: (1) less than one to three times a month, and (2) more than once a week [20]. To assess dietary parameters post-intervention, 24 h dietary recall was used, and for the quantification of nutrients, Food Processor software version 10.11.0 was used.

A dietary antioxidant quality score (DAQs) was used to calculate antioxidant nutrient intake. The score refers to the intake of certain vitamins and minerals that have been proven to act as dietary antioxidants (i.e., selenium, zinc, vitamin A, vitamin C, and vitamin E). Daily nutrient intake was compared to the recommended dietary intake (RDI) for the Mexican population [21]. The intake of each of the five antioxidant nutrients was assessed separately by assigning a value of zero or one to each nutrient. When the intake was below 2/3 of the RDI, it was assigned a value of zero, and when the intake was higher than 2/3 of the RDI, it was assigned a value of one. Thus, the DAQs ranged from zero (very poor quality) to five (high quality) [22].

**Figure 1:**
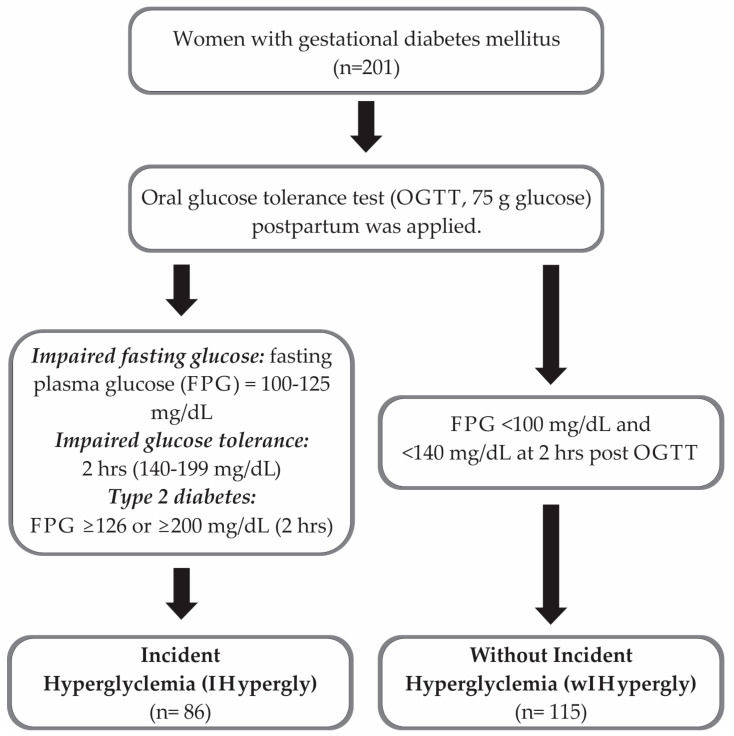
Study flow diagram.

Oxidative stress markers malondialdehyde (MDA) [23], reduced glutathione (GSH) [24], antioxidant capacity (DPPH) [25], and carbonylated proteins [26] were evaluated from serum samples using colorimetric assays, and adiponectin was measured using R&D kits DY1065 (R&D systems, Minneapolis, MN, USA) with a sandwich enzyme-linked immunosorbent assay (ELISA). They were evaluated two times: at baseline (end of pregnancy) and post-intervention (18 months later) (Figure 1).

Participants were excluded from the study if they were not tested with an OGTT after the third month postpartum, if they were taking any medication that modified their glucose, and if they reported intakes of <500 and >5000 kilocalories a day in the food records since it is considered unreliable and inadequate.

Participants accepted and signed informed consent, without economic benefits.

### Statistical Analysis

For the comparison between I-Hypergly vs. wI-Hypergly, a Mann–Whitney U or Student t was used for quantitative variables and Pearson chi-square for categorical variables. A multivariate logistic regression analysis was built to evaluate the variables that affect the outcome. The model was adjusted for age, pregestational BMI, baseline HbA1c, reduced glutathione, malondialdehyde, antioxidant capacity, adiponectin, smoking, DAQs, sugary foods, and carbonylated proteins. Statistical analyses were performed with the statistical program SPSS version 21 (SPSS Inc., Chicago, IL, USA). *p* < 0.05 was considered statistically significant.

## 3. Results

### Description of the Sample

After the glucose tolerance test, 86 patients were classified as incident hyperglycemia. Among them, 6% (*n* = 12) progressed to type 2 diabetes and 36.8% (*n* = 74) to prediabetes. Women with IHypergly had a higher pregestational BMI (Kg/m^2^) (30.37 ± 5.45 vs. wIHypergly 28.47 ± 4.81, *p* = 0.010), and a higher BMI (Kg/m^2^) at the end of pregnancy (32.68 ± 5.20 vs. 31.06 ± 4.23, *p* = 0.016). Age, weight gain during pregnancy, physical activity, smoking status, and vitamin supplementation were not different between groups. Post-intervention, women with IHypergly had a higher BMI (Kg/m^2^) (31.15 ± 5.52 vs. 28.77 ± 4.79, *p* = 0.016). The difference in BMI between post-intervention and pregestational, physical activity, smoking status, and breastfeeding were not statistically different between groups (Table 1).

Women with IHypergly at baseline had a higher HbA1c (%) at 5.85 (5.50–6.12) vs. 5.60 (5.30–5.80), *p* < 0.001. There were no statistically significant differences in fasting plasma glucose, triglycerides, LDL-cholesterol, fasting plasma insulin, and HOMA-IR at baseline, but only LDL-cholesterol remained without differences post-intervention. Post-intervention, women with IHypergly had a higher fasting plasma glucose, higher glucose at 2 h post-OGTT, higher triglycerides, higher fasting insulin, higher insulin at 2 h post-OGTT, higher HbA1c, and higher HOMA-IR (Table 2).

At baseline, women with IHypergly ate sugary foods with more frequency (more than once a week). Energy, proteins, carbohydrates, fats, and DAQs did not show statistically significant differences. Post-intervention, the percentage of total energy from carbohydrates was lower in women with incident hyperglycemia. The dietary parameters of energy, proteins, fats, and DAQs did not show statistically significant differences (Table 3).

Women with GDM with incident hyperglycemia at baseline had higher GSH, MDA, carbonylated proteins, and adiponectin than women without incident hyperglycemia. Antioxidant capacity did not show statistically significant differences. Post-intervention, women with IHypergly had higher GSH, lower MDA, and higher DPPH and adiponectin than wIHypergly women. Carbonylated proteins did not show statistically significant differences (Table 4).

In the multivariate logistic regression analysis in women with GDM, pregestational BMI (Kg/m^2^), HbA1c (%), malondialdehyde, and consumption of sugary foods were associated with an increased risk of developing IHypergly, while the DAQs was associated with a lower risk of incident hyperglycemia. The variables included in the model were age, pregestational BMI, baseline HbA1c, GSH, MDA, DPPH, adiponectin, smoking, breastfeeding, DAQs, sugary foods (more than once a week), and carbonylated proteins Δ. The model had an r = 0.303, and *p* = 0.047 (Table 5).

MDA was the only oxidative stress marker associated with incident hyperglycemia. Therefore, a multivariate analysis was performed to assess which variables had an impact on high MDA levels. There was a directly proportional relationship between GSH (OR 1.141 (1.049–1.241), *p* = 0.002, 95% confidence), DPPH (OR 1.062 (1.037–1.088), *p* < 0.001, 95% confidence), and adiponectin levels (OR 1.001 (1–1.001), *p* < 0.001, 95% confidence) with MDA levels. Also, women who were physically active (moderate and high physical activity) during pregnancy had lower levels of MDA (OR 0.439 (0.212–0.908), *p* = 0.026, 95% confidence).

## 4. Discussion

The present study highlighted differences between GDM women who progressed to T2D and those that did not, which included MDA, and antioxidant capacity, as well as a lack of glycemic control and high consumption of sugary foods.

Women with incident hyperglycemia at baseline had a higher pregestational and end-of-pregnancy BMI, as expected, and this is a risk factor for developing T2D. In fact, women with IHypergly gained 1.24 Kg between pregestational and post-intervention, while the wIHypergly group gained 0.16 Kg and appeared to retain less weight. It is known that retaining weight is also a risk factor for the progression of T2D in women with previous GDM [27]. In addition, IHypergly women breastfed less, which might explain why they lost less weight.

As expected, fasting plasma glucose, glucose at 2 h post-load, triglycerides, fasting plasma insulin, insulin 2 h post-load, HbA1c, and HOMA-IR were higher in incident hyperglycemia.

Women with IHypergly more frequently ate sugary foods (fruit paste, orange juice, honey, marmalade, and flavored water). Although it was not statistically significant, women with incident hyperglycemia ate fewer antioxidants (DAQs), indicating a less healthy diet. An important part of successful lifestyle interventions to prevent T2D in people at risk is eating a healthy diet, with more foods rich in antioxidants like fruit and vegetables, and avoiding sugary foods [15]. Women with IHypergly post-intervention also ate less carbohydrates and more fat. We do not know if those women who progressed did not have a healthy diet throughout their pregnancy, which has been reported as a risk factor for progression to T2D, especially when low-carbohydrate diets are substituted for high animal fat consumption [28].

Women with GDM with IHypergly at baseline had higher GSH, MDA, carbonylated proteins, and adiponectin than wIHypergly women. The MDA and carbonylated proteins were expected as reported elsewhere [8,9]. However, GSH and adiponectin seem inverted; this could be due to compensation for elevated oxidative stress during pregnancy. Post-intervention, women with IHypergly had higher GSH, DPPH, and adiponectin, and lower MDA, which was unexpected. This suggests that the disease has started, and that women are fighting oxidative stress and again compensating for it. A recent article proposes that mitochondrial dysfunction in GDM increases oxidative stress and impairs the insulin signaling pathway, which would increase the risk of hyperglycemia [29]. Inflammation related to obesity has long been recognized as a factor for oxidative stress [30]. In the present study, women that progressed to hyperglycemia were obese, unlike those who did not.

In the multivariate logistic regression analysis, we can see those women with more pregestational BMI, HbA1c, MDA, and who consumed sugary foods, and had less intake of antioxidants had a higher risk of incident hyperglycemia after GDM.

Higher MDA levels were positively associated with GSH, DPPH, and adiponectin. This might be explained by the fact that there is more oxidative stress, so GSH, DPPH, and adiponectin are trying to compensate for it. These three markers are antioxidants and anti-inflammatory.

Women who performed physical activity during pregnancy had lower levels of MDA. This goes in accordance with a case–control study of 64 participants with T2D, where 31 performed 12 weeks of aerobic exercise training (three times per week) and 33 performed no exercise, only normal daily activities (control). MDA levels decreased (*p* < 0.05) in the active group [31]. In another study on T2D (*n* = 75), participants who performed moderate physical activity and who were physically active had lower levels of MDA and total antioxidant capacity (*p* < 0.05) [32]. Exercise has many benefits that could explain the decrease in MDA levels because by improving insulin sensitivity and glucose control, and lowering insulin resistance, oxidative stress is reduced [33]. However, after the intervention, most women returned to their pre-pregnancy activities, without time for increased physical activity.

In our population, in the second logistic model, there were no statistically significant differences between women with and without GDM and MDA levels. This might be because women with GDM had subtle hyperglycemia, and MDA responds to it. Also, women with GDM in our cohort were well-controlled during pregnancy [8]. A third possibility is that some GDM women had undiagnosed prediabetes or T2D.

The present study had several strengths, including the fact that, to our knowledge, this is one of the few studies evaluating the association between oxidative stress markers with incident hyperglycemia in women with gestational diabetes mellitus, as well as reporting the association between lower MDA levels and being physically active during pregnancy.

However, there are some limitations that should be recognized. There may be measurement errors and reporting bias inherent to the FFQ and 24 h dietary recall, such as over- or underreporting of food consumption in general or of specific foods, and estimations of portion size as they are affected by the beliefs and nutritional knowledge of the patients.

## 5. Conclusions

Malondialdehyde is associated positively with incident hyperglycemia, and negatively with antioxidant capacity. In addition, a lack of glycemic control and/or high consumption of sugary foods contributed to the progression to incident hyperglycemia. Timely identification of these factors could help avoid progression to hyperglycemia and type 2 diabetes. Therefore, we suggest follow-up studies on the oxidant and antioxidant status of patients with GDM to prevent long-term complications in the mother.

## Figures and Tables

**Table 1:** Clinical and anthropometric parameters among women with vs. without incident hyperglycemia at baseline and post-intervention.

	*Baseline*			*Post-Intervention*		
	IHypergly (*n* = 86) Median (IQR)	wIHypergly (*n* = 115) Median (IQR)	*p*	IHypergly (*n* = 86) Median (IQR)	wIHypergly (*n* = 115) Median (IQR)	*p*
Age (years)	33 (31–38)	34 (30–37)	0.402	___	___	___
Pregestational and post-intervention BMI (Kg/m^2^) *	30.37 ± 5.45	28.47 ± 4.81	**0.010**	31.15 ± 5.52	28.77 ± 4.79	**0.016**
BMI Δ (post-intervention-pregestational) (Kg/m^2^) *	___	___	___	0.77 ± 2.91	0.32 ± 2.89	0.570
End of pregnancy BMI (Kg/m^2^) *	32.68 ± 5.20	31.06 ± 4.23	**0.016**	**___**	**___**	**___**
Weight gained during pregnancy (Kg) *	5.88 ± 6.10	6.38 ± 6.66	0.587	___	___	___
Systolic blood pressure (mm Hg) *				116.57 ± 12.54	113.93 ± 10.44	0.206
Diastolic blood pressure (mm Hg) *				75.96 ± 9.50	73.86 ± 8.58	0.197
Physically active, *n* (%) ^	55 (64)	83 (73.5)	0.150	41 (47.7)	59 (51.3)	0.611
Actively smoking, *n* (%) ^	10 (11.8)	11 (9.6)	0.616	10 (11.6)	14 (12.2)	0.906
Vitamin supplementation, *n* (%) ^	66 (76.7)	89 (77.4)	0.914	___	___	___
Gestational age (weeks)	38.00 (37.05–39)	38.30 (38–39)	0.103			
Apgar score	9 (9–9)	9 (9–9)	0.862			
Breastfeeding, *n* (%) ^	___	___	___	44 (51.2)	73 (63.5)	0.080

Mann–Whitney U, * Mean (SD), Student t; ^ Pearson Chi-Square, *p* < 0.05 IHypergly: incident hyperglycemia; wIHypergly: without incident hyperglycemia; BMI: body mass index; physically active per IPAQ manual.

**Table 2:** Biochemical parameters among women with GDM with vs. without incident hyperglycemia at baseline and post-intervention.

	*Baseline*			*Post-Intervention*		
	IHypergly (*n* = 86) Median (IQR)	wIHypergly (*n* = 115) Median (IQR)	*p*	IHypergly (*n* = 86) Median (IQR)	wIHypergly (*n* = 115) Median (IQR)	*p*
Fasting plasma glucose (mg/dL)	69 (59–83.50)	70 (61.75–85.25)	0.866	101 (94–108.25)	86 (77–93)	**<0.001**
Glucose 2 h (mg/dL) post OGTT	___	___	___	143 (113–169)	96 (86–117)	**<0.001**
Triglycerides (mg/dL)	348 (282.50–429.50)	327 (277–410)	0.163	153 (106.75–219)	125 (86–174)	**0.001**
LDL-cholesterol (mg/dL) *	138.45 ± 43.04	141.38 ± 37.59	0.609	122.73 ± 30.68	113.08 ± 30.05	0.929
Fasting plasma Insulin (μU/mL)	13.10 (8.32–25.70)	10.40 (6.82–18.72)	0.072	11.60 (7.60–17.60)	7.60 (5.10–10.30)	**<0.001**
Insulin 2 h (μU/mL) post OGTT	___	___	___	60.75 (36.15–94.27)	31.55 (16.92–54.85)	**<0.001**
HbA1c (%)	5.85 (5.50–6.12)	5.60 (5.30–5.80)	**<0.001**	5.8 (5.50–6.10)	5.50 (5.20–5.70)	**<0.001**
HOMA-IR	2.13 (1.40–5.30)	1.82 (1.10–4.12)	0.196	2.98 (1.77–4.86)	1.53 (1.02–2.25)	**<0.001**

Mann–Whitney U, * Mean (SD), Student t; *p* < 0.05 IHypergly: incident hyperglycemia; wIHypergly: without incident hyperglycemia; OGTT: oral glucose tolerance test; HbA1c: glycated hemoglobin A1c.

**Table 3:** Dietary parameters among women with GDM with vs. without incident hyperglycemia at baseline and post-intervention.

	*Baseline*			*Post-Intervention*		
	IHypergly (*n* = 86) Median (IQR)	wIHypergly (*n* = 115) Median (IQR)	*p*	IHypergly (*n* = 86) Median (IQR)	wIHypergly (*n* = 115) Median (IQR)	*p*
Energy (Kcals)	1836.82 (1489.44–2330.01)	2008.68 (1623.69–2396.22)	0.237	1694.83 (1370.53–2183.88)	1788.55 (1406.18–2307.81)	0.374
Proteins (% TE)	13.89 (12.76–15.55)	14.31 (13.32–15.81)	0.196	18.45 (15.59–22.93)	17.93 (14.46–21.35)	0.359
Carbohydrates (% TE) *	50.94 ± 6.79	50.36 ± 6.78	0.550	47.84 ± 10.57	51.06 ± 10.32	**0.032**
Fats (% TE) *	36.41 ± 5.67	37 ± 6.36	0.496	33.08 ± 11.84	30.83 ± 9.74	0.105
Sugary foods (more than once a week), *n* (%) ^	36 (41.9)	32 (27.8)	**0.037**	___	___	___
DAQs, 0 (very poor quality) to 5 (high quality) ^	3 (2–4.25)	4 (3–5)	0.131	2 (1–3)	2 (1–3)	0.401

IHypergly: incident hyperglycemia; wIHypergly: without incident hyperglycemia; TE: total energy; DAQs: daily antioxidant quality score, Mann–Whitney U, * Mean (SD), Student t; ^ Pearson Chi-Square, *p* < 0.05.

**Table 4:** Oxidative stress markers among women with GDM with vs. without incident hyperglycemia at baseline and post-intervention.

	*Baseline*			*Post-Intervention*		
	IHypergly (*n* = 86) Median (IQR)	wIHypergly (*n* = 115) Median (IQR)	*p*	IHypergly (*n* = 86) Median (IQR)	wIHypergly (*n* = 115) Median (IQR)	*p*
Reduced glutathione (GSH) (µM)	11.93 (10.50–15.08)	11.45 (8.83–13.83)	**0.018**	14.52 (12.85–16.21)	12.17 (8.71–14.55)	**<0.001**
Malondialdehyde (MDA) (nmol)	35.81 (33.77–39.28)	34.69 (13.57–37.96)	**0.001**	11.63 (10.81–38.98)	40.51 (36.94–55.10)	**<0.001**
Antioxidant capacity (DPPH) (%)	39.86 (33.29–46.41)	40.71 (32.62–49.22)	0.500	41.98 (36.37–52.91)	40.00 (33.90–46.38)	**0.007**
Carbonylated proteins (nmol/mL)	34.31 (28.97–40.96)	31.59 (27.04–36.59)	**0.033**	29.54 (24.43–33.92)	30.68 (26.59–34.54)	0.096
Adiponectin (pg/mL)	3993.77 (3371.17–4334.83)	3620.53 (2746.84–4054.90)	**0.001**	3133.03 (2805.04–3369.43)	2868.38 (2570.18–3183.28)	**0.001**

Mann–Whitney U, *p* < 0.05 IHypergly: incident hyperglycemia; wIHypergly: without incident hyperglycemia.

**Table 5:** Multivariate logistic regression analysis in women with GDM and incident hyperglycemia.

	RR	95% CI	*p*
Pregestational BMI (kg/m^2^)	1.085	1.015–1.161	**0.017**
HbA1c (%)	3.103	1.550–6.213	**0.001**
Malondialdehyde (nmol)	1.033	1.006–1.060	**0.015**
DAQs	0.776	0.607–0.991	**0.042**
Sugary foods (more than once a week)	2.221	1.069–4.615	**0.032**

Variables of the model: age, pregestational BMI, baseline: HbA1c, reduced glutathione, malondialdehyde, DPPH, adiponectin, smoking, DAQs (dietary antioxidant quality score), breastfeeding, sugary foods, and carbonylated proteins (Δ). Model: r = 0.303, *p* = 0.047.

## Data Availability

The data presented in this study are available on request from the corresponding author due to the fact that the study is still ongoing and sharing the database could affect completion of the trial.

## References

[1] American Diabetes Association (2025). Chapter 2: Diagnosis and Classification of diabetes: Standards of Care in Diabetes-2025. Diabetes Care.

[2] Wang H., Li N., Chivese T., Werfalli M., Sun H., Yuen L., Hoegfeldt C.A., Powe C.E., Immanuel J., Karuranga S. (2022). IDF Diabetes Atlas: Estimation of global and regional gestational diabetes mellitus prevalence for 2021 by International Association of Diabetes in Pregnancy Study Group`s Criteria. Diabetes Res. Clin. Pract..

[3] Campos-Nonato I., Galván-Valencia O., Hernández-Barrera L., Oviedo-Solis C., Barquera S. (2023). Prevalence of obesity and associated risk factors in Mexican adults: Results of the Ensanut 2022. Salud Publica Mex..

[4] Basto-Abreu A., López-Olmedo N., Rojas-Martínez R., Aguilar-Salinas C.A., Moreno-Banda G.L., Carnalla M., Rivera J.A., Romero-Martinez M., Barquera S., Barrientos-Gutiérrez T. (2023). Prevalence of prediabetes and diabetes in Mexico. Ensanut 2022. Salud Publica Mex..

[5] Lappas M., Hidden U., Desoye G., Froehlich J., Hauguel-de Mouzon S., Jawerbaum A. (2011). The role of oxidative stress in the pathophysiology of gestational diabetes mellitus. Antioxid. Redox Signal..

[6] Evans J.L., Goldfine I.D., Maddux B.A., Grodsky G.M. (2003). Are oxidative stress activated signaling pathways mediators of insulin resistance and beta-cell dysfunction?. Diabetes.

[7] Valko M., Leibfritz D., Moncol J., Cronin M.T.D., Mazur M., Telser J. (2007). Free radicals and antioxidants in normal physiological functions and human disease. Int. J. Biochem. Cell Biol..

[8] Zhang C., Yang Y., Chen R., Wei Y., Feng Y., Zheng W., Liao H., Zhang Z. (2019). Aberrant expression of oxidative stress related proteins affects the pregnancy outcome of gestational diabetes mellitus patients. Am. J. Transl. Res..

[9] Li H., Yin Q., Li N., Ouyang Z., Zhong M. (2016). Plasma markers of oxidative stress in patients with gestational diabetes mellitus in the second and third trimester. Obstet. Gynecol. Int..

[10] Saucedo R., Ortega-Camarillo C., Ferreira-Hermosillo A., Díaz-Velázquez M.F., Meixueiro-Calderón C., Valencia-Ortega J. (2023). Role of oxidative stress and inflammation in gestational diabetes mellitus. Antioxidants.

[11] Pheiffer C., Dias S., Jack B., Malaza N., Adam S. (2021). Adiponectin as a potential biomarker for pregnancy disorders. Int. J. Mol. Sci..

[12] Xu J., Zhao Y.H., Chen Y.P., Yuan X.L., Wang J., Zhu H., Lu C.M. (2014). Maternal circulating concentrations of tumor necrosis factor-alpha, leptin, and adiponectin in gestational diabetes mellitus: A systematic review and meta-analysis. Sci. World J..

[13] Li Z., Cheng Y., Wang D., Chen H., Chen H., Ming W.-K., Wang Z. (2020). Incidence rate of type 2 diabetes mellitus after gestational diabetes mellitus: A systematic review and meta-analysis of 170,139 women. J. Diabetes Res..

[14] Bengston A.M., Ramos S.Z., Savitz D.A., Werner E.F. (2021). Risk factors for progression from gestational diabetes to postpartum type 2 diabetes: A review. Clin. Obstet. Gynecol..

[15] Uusitupa M., Khan T.A., Viguiliouk E., Kahleova H., Rivellese A.A., Hermansen K., Pfeiffer A., Thanopoulou A., Salas-Salvadó J., Schwab U. (2019). Prevention of type 2 diabetes by lifestyle changes: A systematic review and meta-analysis. Nutrients.

[16] Li N., Yang Y., Cui D., Li C., Ma R.C., Li J., Yang X. (2021). Effects of lifestyle intervention on long-term risk of diabetes in women with prior gestational diabetes: A systematic review and meta-analysis of randomized controlled trials. Obes Rev..

[17] Aroda V.R., Christophi C.A., Edelstein S.L., Zhang P., Herman W.H., Barrett-Connor E., Delahanty L.M., Montez M.G., Ackermann R.T., Zhuo X. (2015). Diabetes Prevention Program Research Group. The effect of lifestyle intervention and metformin on preventing or delaying diabetes among women with and without gestational diabetes: The Diabetes Prevention Program outcomes study 10-year follow up. J. Clin. Edocrinol. Metab..

[18] Huvinen E., Koivusalo S.B., Meinilä J., Valkama A., Tiitinen A., Rönö K., Stach-Lempinen B., Eriksson J.G. (2018). Effects of a lifestyle intervention during pregnancy and first postpartum year: Findings from the RADIEL study. J. Clin. Endocrinol. Metab..

[19] Craig C.L., Marshall A.L., Sjöström M., Bauman A.E., Booth M.L., Ainsworth B.E., Pratt M., Ekelund U.L., Yngve A., Sallis J.F. (2003). International physical activity questionnaire: 12-country reliability and validity. Med. Sci. Sports Exerc..

[20] Hernández-Avila M., Romieu I., Parra S., Hernández-Avila J., Madrigal H., Willett W. (1998). Validity and reproducibility of a food frequency questionnaire to assess dietary intake of women living in Mexico City. Salud Pública Mex..

[21] Bourges H., Casanueva E., Rosado J.L. (2005). Recomendaciones de Ingestión de Nutrimentos para la Población Mexicana.

[22] Rivas A., Romero A., Mariscal-Arcas M., Monteagudo C., López G., Lorenzo M.L., Ocaña-Peinado F.M., Olea-Serrano F. (2012). Association between antioxidant quality score (DAQs) and bone mineral density in Spanish women. Nutr. Hosp..

[23] Jentzsch A.M., Bachmann H., Fürst P., Biesalski H.K. (1996). Improved analysis of malondialdehyde in human body fluid. Free Radic. Biol. Med..

[24] Tietze F. (1969). Enzymic method for quantitative determination of nanogram amounts of total and oxidized glutathione: Applications to mammalian blood and other tissues. Anal. Biochem..

[25] Brand-Williams W., Cuvelier M.E., Berset C. (1995). Use of a free radical method to evaluate antioxidant activity. Lwt Food Sci. Technol..

[26] Levine R.L., Garland D., Oliver C.N., Amici A., Climent I., Lenz A.G., Ahn B.W., Shaltiel S., Stadtman E.R. (1990). Determination of carbonyl content in Oxidatively modified proteins. Methods Enzymol..

[27] Bao W., Yeung E., Tobias D.K., Hu F.B., Vaag A.A., Chavarro J.E., Mills J.L., Grunnet L.G., Bowers K., Ley S.H. (2015). Long-term risk of type 2 diabetes mellitus in relation to BMI and weight change among women with a history of gestational diabetes mellitus: A prospective cohort study. Diabetologia.

[28] Bao W., Li S., Chavarro J.E., Tobias D.K., Zhu Y., Hu F.B., Zhang C. (2016). Low carbohydrate-diet scores and long-term risk of type 2 diabetes among women with history of gestational diabetes mellitus: A prospective cohort study. Diabetes Care.

[29] Torres-Torres J., Monroy-Muñoz I.E., Perez-Duran J., Solis-Paredes J.M., Camacho-Martinez Z.A., Baca D., Espino-Y-Sosa S., Martinez-Portilla R., Rojas-Zepeda L., Borboa-Olivares H. (2024). Cellular and Molecular Pathophysiology of Gestational Diabetes. Int. J. Mol. Sci..

[30] Martinez-Martinez E., Cachofeiro V. (2022). Oxidative stress in obesity. Antioxidants.

[31] Arslan M., Ipekci S.H., Kebapcilar L., Dede N.D., Kurban S., Erbay E., Gonen M.S. (2014). Effect of Aerobic Exercise Training on MDA and TNF-alpha Levels in Patients with Type 2 Diabetes Mellitus. Int. Sch. Res. Not..

[32] Alghadir A.H., Gabr S.A., Anwer S., Al-Eisa E. (2016). Fatigue and Oxidative Stress Response to Physical Activity in Type 2 Diabetic Patients. Int. J. Diabetes Dev. Ctries..

[33] Małkowska P. (2024). Positive Effects of Physical Activity on Insulin Signaling. Curr. Issues Mol. Biol..

